# Mode of delivery affected questionnaire response rates in a birth cohort study

**DOI:** 10.1016/j.jclinepi.2016.09.004

**Published:** 2017-01

**Authors:** Isabelle Bray, Sian Noble, Ross Robinson, Lynn Molloy, Kate Tilling

**Affiliations:** a Department of Health and Social Science, University of the West of England, Frenchay Campus, Bristol BS16 1QY, England; b School of Social and Community Medicine, University of Bristol, Canynge Hall, 39 Whatley Road, Bristol BS8 2PS, England; c ALSPAC, School of Social and Community Medicine, University of Bristol, Oakfield House, Oakfield Grove, Bristol BS8 2BN, England

**Keywords:** Randomized controlled trial, Online questionnaire, Response rates, Cohort study, ALSPAC, Mixed mode

## Abstract

**Objectives:**

Cohort studies must collect data from their participants as economically as possible, while maintaining response rates. This randomized controlled trial investigated whether offering a choice of online or paper questionnaires resulted in improved response rates compared with offering online first.

**Study Design and Setting:**

Eligible participants were young people in the Avon Longitudinal Study of Parents and Children (ALSPAC) study (born April 1, 1991, to December 31, 1992, in the Avon area). After exclusions, 8,795 participants were randomized. The “online first” group were invited to complete the questionnaire online. The “choice” group were also sent a paper questionnaire and offered a choice of completion method. The trial was embedded within routine data collection. The main outcome measure was the number of questionnaires returned. Data on costs were also collected.

**Results:**

Those in the “online first” arm of the trial were less likely to return a questionnaire [adjusted odds ratio: 0.90; 95% confidence interval (CI): 0.82, 0.99]. The “choice” arm was more expensive (mean difference per participant £0.71; 95% CI: £0.65, £0.76). It cost an extra £47 to have one extra person to complete the questionnaire in the “choice” arm.

**Conclusion:**

Offering a choice of completion methods (paper or online) for questionnaires in ALSPAC increased response rates but was more expensive than offering online first.

**What is new?**

**Key findings**
• In this birth cohort study, offering a choice of online/paper questionnaire (concurrent mixed mode) resulted in higher response rates than offering online-only first (sequential mixed mode).

**What this adds to what was known?**
• Cohort studies administering questionnaires should weigh this benefit in terms of response rates against the increase in cost associated with offering a choice.

**What is the implication and what should change now?**
• This trial should be replicated in other cohorts of different ages, considering the effects in different demographic subgroups.

## 1 Introduction

As the fields of lifecourse epidemiology and epigenetics develop, multigenerational birth cohort studies are becoming increasingly important to health and social research [1], [2]. Initial response rates to population cohort studies have decreased over recent decades, and such studies experience declining participation rates throughout the lifetime of the study [3]. Selection and attrition bias therefore threaten the validity and viability of large cohort studies. Reasons for attrition are generally divided into (1) failure to locate (i.e., address changes), (2) failure to contact, and (3) refusal to participate. There is a considerable literature around the best methods to keep up-to-date addresses for study participants, often referred to as “tracking” (e.g., [4], [5], [6]). Failure to contact is most relevant to studies which seek face-to-face contact for data collection (e.g., the UK Household Longitudinal Survey). Maximizing participation, whether that be participating in an interview, attending a clinic, or completing a questionnaire, is crucial to the success of any cohort study. The approach taken by individual cohort studies is usually based on shared experience of best practice [7], although randomized controlled trials (RCTs) are increasingly being used to assess methods to improve response rates in cohort studies, for example, Boyd et al. [8]. Booker et al. [9] carried out a systematic review of various retention methods used by population cohort studies and concluded that incentives boost retention, but that the other methods they assessed (e.g., reminders, alternative methods of data collection) had not been sufficiently evaluated. Only 11 (39%) of the 28 studies included in the review were RCTs of methods for cohort retention. This highlights the lack of evidence about which cohort retention methods are most effective. There is better evidence about measures to improve response rates to questionnaires [10]. For example, the use of incentives has been shown to improve response rates to electronic health surveys [11].

The Avon Longitudinal Study of Parents and Children (ALSPAC) is a birth cohort study which is following up children born in a 21-month period in 1991–1992. Questionnaire data in ALSPAC have traditionally been collected by postal questionnaires. For large cohort studies, particularly in times of austerity, online data collection is a financially attractive option [12]. It is also assumed to appeal to younger participants, whom have grown up in an electronic age and for whom mobile devices and social media are integral to their lives [13]. Although online methods have been used for some data collection exercises in ALSPAC, the reported response rates [14] suggest that participants are not ready to move to an online-only model, and the main questionnaires until 2012 were all administered on paper. But, like some other cohort studies, for example, Growing up Today (http://www.gutsweb.org/), ALSPAC is seeking to move its participants toward online questionnaire completion for a variety of reasons. The main drivers are presumed improved response rates and reduced costs. The online approach is also expected to speed up the process of questionnaire administration and data entry, improve data accuracy, and reduce environmental costs. The anticipated improvements from the participants' point of view include choice about how and when to complete the questionnaire (particularly as functionality on Smartphones improves) and instant and easy submission of data (reducing unwanted reminders and the need to find a postbox).

However, there are concerns about using an online-only approach for data collection. Evidence from both market research [15] and health-related research [16], [17] suggests that it will lead to lower response rates than traditional paper questionnaires. Furthermore, online data collection could exacerbate the bias toward more educated participants that typically arises through selection and attrition in cohort studies, as those with less access to the Internet might be discouraged from taking part [18], [19]. In practice, however, online data collection in cohort studies is likely to be followed up with an option to complete a paper version (a sequential mixed-mode approach). The use of multiple methods of data collection in surveys has been debated [20] and may even reduce response rates [21] but is reported to have the potential to achieve similar response rates to those of traditional postal questionnaires [22]. There is ongoing discussion in the survey literature about the relative merits of concurrent and sequential mixed-mode approaches [23], with some authors suggesting that a sequential approach is superior [24]. At the same time, there is a paucity of evidence comparing concurrent and sequential mixed-mode approaches in population cohort studies administering lengthy questionnaires [19]. We have conducted a nested RCT comparing an “online first” (sequential mixed mode) arm with a “choice” (concurrent mixed mode) arm, in a routine follow-up of a birth cohort (aged 21 years at the time of the RCT). We compared the two approaches in terms of response rates, completion rates, and costs, including administrative time.

## 2 Methods

### 2.1 ALSPAC cohort

ALSPAC recruited 14,541 pregnant women resident in Avon, UK, with expected dates of delivery April 1, 1991, to December 31, 1992; 14,541 is the initial number of pregnancies for which the mother enrolled in the ALSPAC study and had either returned at least one questionnaire or attended a “Children in Focus” clinic by July 19, 1999. Of these initial pregnancies, there was a total of 14,676 fetuses, resulting in 14,062 live births and 13,988 children who were alive at 1 year of age.

When the oldest children were approximately 7 years of age, an attempt was made to bolster the initial sample with eligible cases who had failed to join the study originally. As a result, when considering variables collected from the age of 7 years onward (and potentially abstracted from obstetric notes), there are data available for more than the 14,541 pregnancies mentioned above. Further phases of enrollment are described in more detail in the cohort profile [14].

The total sample size for analyses using any data collected after the age of 7 years is therefore 15,247 pregnancies, resulting in 15,458 fetuses. Of this total sample of 15,458 fetuses, 14,775 were live births and 14,701 were alive at 1 year of age. These children, now young people (YP) of around 24 years of age, and their parents (or step parents) have been followed in detail until the present day. Please note that the study website contains details of all the data that is available through a fully searchable data dictionary (http://www.bristol.ac.uk/alspac/researchers/data-access/data-dictionary/).

### 2.2 Subjects

We devised a parallel-group RCT for the 2012 YPs' questionnaire [called “It's All About You (20+)”]. The study participants included in this data collection exercise are all YPs eligible to receive the questionnaire planned for 2012. Exclusions were due to death, withdrawal from the study, a noncontact status flag on the study database (e.g., due to family problems or bereavement), because returned mail has previously indicated that ALSPAC does not have the correct address, or because they have opted out of questionnaires. The “online first” arm of the trial received a letter with a link to the online questionnaire. The “choice” arm of the trial also received a letter with a link to the online questionnaire, in addition to a paper copy of the questionnaire (with prepaid return envelope) so that a choice of method was offered. Participants were not aware of the trial, as it was carried out as part of routine data collection.

Assuming an expected response rate of 50% (based on previous YP questionnaires), it was calculated that 1,605 participants in each arm of the trial would have 80% power to detect a difference in response rates of 5% between the two arms.

### 2.3 Paper questionnaire

The paper version of the questionnaire was an A5 booklet of 44 pages. Excluding the three administrative questions at the end, there were five sections: “Children of the 90s”; “Gambling”; “Deliberate Self-Harm”; “Employment, Education and Training”; and “Tobacco and Alcohol.” The number of questions in each section ranged from 7 to 43 (median 17), but many of these questions had several parts. Skip statements were used to divert participants around questions that were not relevant to them. The number of questions in each section that were followed by a skip statement ranged from 1 to 9 (median 5).

### 2.4 Online questionnaire

The online questionnaire was designed to be as similar as possible to the paper questionnaire, acknowledging that certain functions, such as skip statements, would be different because participants would be automatically directed to the next relevant question. This also affected the numbering of questions, which would have been nonconsecutive if not allowed to be dynamic in the online version.

Generally, the number of questions per page was less in the online version than in the paper version. The ability to have a variable page length was one of the key differences between paper and online as it allowed more logical grouping of questions. Finally, for online completion, an approximate progress indicator bar was given for each section.

### 2.5 Reminders and compensation

The reminder schedule was the same for both arms of the trial (Table 1). The first reminder (after 3 weeks) was by email, but if an email address was not recorded and a mobile number was on record, then a text was used. If neither electronic means of contact was possible, then a postcard reminder was used. Two weeks later, a different mode of reminder was sent (unless a postcard had already been used in which case no other reminder was sent, to avoid multiple reminders of the same method). Eight weeks after the initial letter, a reminder letter was sent to all nonrespondents, with a paper copy of the questionnaire enclosed. Another brief reminder (email, text, or postcard) was sent if necessary 2 weeks later. A Facebook reminder was also posted 12 weeks after the original letter. Finally, a phone call reminder was attempted for all those who had not responded between 12 and 19 weeks after the initial letter was sent out. Initially, an attempt was made to contact the participant using the landline number held on record. If this was not successful and if a mobile number was also recorded, then this number was also rung. If neither attempt was successful, then a message was left on both landline and mobile phones, wherever possible. If contact was made with a family member but the participant was not at home, then a message was left.

A reminder was sent only if a paper questionnaire had not been received from the participant and the online submission was not complete (i.e., at least one section of the questionnaire had not been submitted online). The exception to this was the reminder at 8 weeks enclosing a paper questionnaire—this was only sent if a paper questionnaire had not been received and an online submission had not been initiated (i.e., no sections had been submitted online). If a participant contacted ALSPAC to request a paper questionnaire at any stage in the process, then this was recorded and one was sent.

A £10 Amazon voucher was offered to compensate participants for their time and to encourage response.

### 2.6 Pilot study

To test that both arms of the trial and the reminder process, which was more intensive than any used previously in ALSPAC, were acceptable to participants, and to identify any logistical problems, the RCT was piloted on 200 participants. To maximize the efficiency of the pilot study, we chose participants with a high probability of responding. They were randomly chosen from among those who had responded to the YP questionnaire administered around age 18 years. These YPs were then assigned to each arm of the trial using simple randomization. Because it was important to complete the pilot quickly, to administer the main questionnaire on schedule, the reminders were issued at 2 weekly intervals rather than the timeframe set out in Table 1. This was not felt to detract from the usefulness of the pilot study in testing the acceptability of the strategy as, if anything, more frequent reminders would be received less positively by participants.

### 2.7 Randomization

The remaining YPs were randomly assigned with equal probability to either the “online first” arm or the “choice” arm. Randomization was stratified on important confounders—national tertiles of Index of Multiple Deprivation (IMD) score 2010, based on postcode [25], gender, and level of participation (<90% or ≥90% participation calculated over the course of the study). The 200 participants in the pilot phase were excluded.

Randomization of participants was carried out using the runiform function in Stata with anonymous identifiers. This was performed by a researcher who was not involved with the ALSPAC administration process. The results of the randomization were passed to the questionnaire administration team, who implemented the mailings and reminders for both arms of the trial.

### 2.8 Outcome measures

The primary outcome measure was the number of questionnaires returned (with at least one question answered) in each arm of the trial. For the purposes of this analysis, return rates were calculated 30 weeks after the initial mailing.

A secondary outcome was completeness of questionnaires. For returned questionnaires, the number of questions answered was compared, to see if either approach (online first or choice of online/paper questionnaire) encouraged more complete responses. ALSPAC staff compiling the completion statistics were blinded to group assignment. Of the 314 possible questions included in the full questionnaire, 109 of these were not dependent on skip statements, and the analysis of completeness was repeated for this core subset of questions.

Other secondary outcomes included: mode of response (online or paper); time taken to respond; the number of reminders issued; and requests for paper questionnaires.

Resources used in the administration of each arm of the trial were identified. These included: printing, packing and posting of letters and questionnaires (including address labels and envelopes); printing and posting of reminder postcards; and text and phone call reminders. The amount of administrative time spent on individual calls and texts was based on an average for a sample of these communications spread over time.

Costs were applied to these resources. Administration time was valued using the wage rate per minute for casual staff. Consumables (e.g., printing, labels, paper, postage) were valued on a per item basis. Resources were only compared where there was a difference in costs between the two arms. Setup costs, for example, creation of database, design of questionnaire, and design of the data entry form, were not included as both the online version and the paper-based version had to be available for both arms in this trial. Some of the higher level administration costs (which included the time to send emails) for each stage of the reminder processes were also excluded as they were the same across both arms. The actual costs of telephone calls were also excluded because business telephone packages are based on a monthly fee.

### 2.9 Analysis

Analysis was carried out on an intention-to-treat basis. Logistic and robust multiple linear regression was used to compare responses, and other secondary outcomes, between the two arms of the trial, adjusting for stratification variables (gender, previous participation score, IMD tertile).

The mean cost per participant for each item of resource use for the two arms of the trial was calculated as the mean resource use per participant multiplied by the unit cost for that resource. The total mean cost per participant for the two arms of the trial was then calculated by summing up the individual cost items and dividing by the number of participants in each arm. An estimate of the extra cost to have one extra person to complete a questionnaire was calculated as the difference in total costs between the two arms divided by the difference in the number of people who completed a questionnaire, defined as completed at least one question.

### 2.10 Ethical approval

Ethical approval was obtained from the ALSPAC Ethics and Law Committee (ref: E201215).

## 3 Results

### 3.1 Pilot

Overall, response rates were very similar in both arms of the pilot trial—84% in the “online first” arm and 81% in the “choice” arm—with no evidence of a difference (*P* = 0.6), suggesting that the planned RCT would not have a detrimental effect on response rates. These percentages are based on 100 participants in each arm and a cutoff of approximately 24 weeks after the initial mailing. As expected, the number of responses that were submitted online was greater in the “online first” arm (69) than in the “choice” arm (37). ALSPAC monitored feedback from participants and concluded that both arms of the trial and the reminder process were acceptable to participants.

### 3.2 Numbers of participants in the RCT

**Fig. 1:**
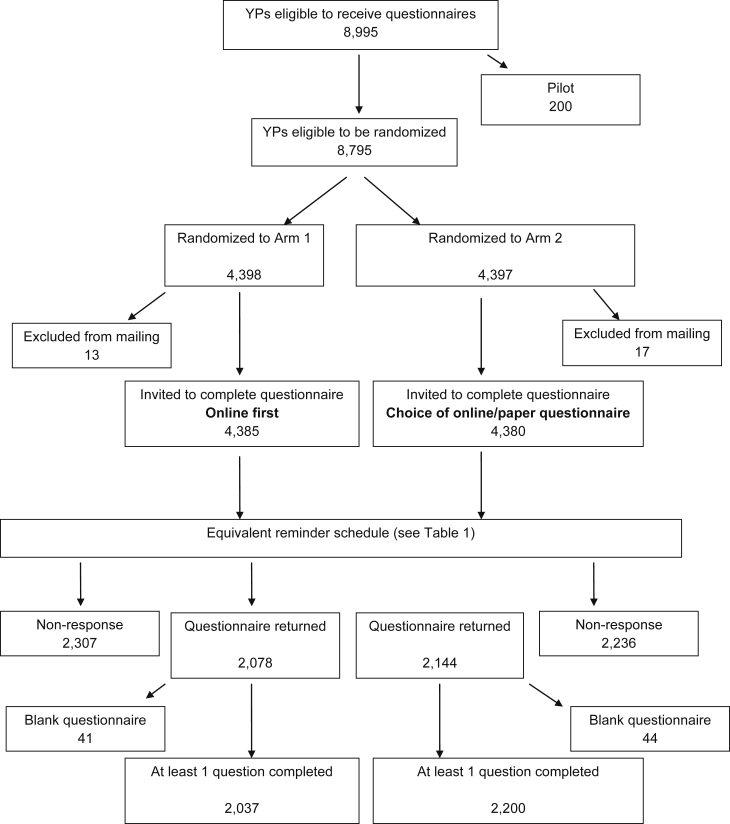
Flow diagram of participant numbers. YP, young people.

A flow diagram of participant numbers in the RCT is shown in Fig. 1. Excluding those in the pilot exercise and who could not be included for the other reasons listed in the Section 2, a total of 8,795 participants were available for randomization, exceeding the required sample size. Of these, 329 (3.7%) had missing IMD score so were randomly assigned to an IMD category (tertile). The stratified randomization resulted in an equal number of participants in each arm of the trial (4,398 in the “online first” arm and 4,397 in the “choice” arm) and an equal distribution of stratification variables in each arm (49% male, 19% most deprived tertile, 50% least deprived tertile, 67% with a lower than 90% participation score). Before participants were mailed, a final check was made of their status flags on the study database. It was discovered that 13 participants in the “online first” arm (0.3%) and 17 in the “choice” arm (0.4%) were not eligible (e.g., due to changes in family circumstances or requests not to be contacted) and were therefore not mailed, but were included in this analysis, which was conducted on an intention-to-treat basis. Conversely, some participants who were not originally randomized went on to complete a questionnaire, but are not included in the analysis. The numbers of reminders sent at each reminder stage are shown in Supplementary Table 1 at www.jclinepi.com. At each stage, and for each mode (e.g., email, text, postcard), more reminders were sent in the “online first” arm (*P* < 0.05).

### 3.3 Primary and secondary outcome measures

Table 2 shows the number of questionnaires returned in each arm of the trial. Having adjusted for the stratification variables, participants in the “online first” arm were 10% less likely to return a questionnaire than those offered a choice [adjusted odds ratio = 0.90; 95% confidence interval (CI): 0.82, 0.99; *P* = 0.04].

The mean time to respond (by postal questionnaire or a section submitted online) was 3.5 days shorter in the “online first” arm than in the “choice” arm of the trial (95% CI: 1.2, 5.9; *P* = 0.003). Other secondary outcomes are shown in Supplementary Tables 2 and 3 at www.jclinepi.com.

### 3.4 Economic analysis

A detailed economic breakdown for each arm of the trial is given in Supplementary Table 4 at www.jclinepi.com, including the costs associated with each type of reminder. The mean cost per participant was £3.14 (95% CI: £3.10, £3.18) in the “choice” arm and £2.43 (95% CI: £2.39, £2.47) in the “online first” arm. Adjusting for the stratification variables, this led to a mean difference per participant of £0.71 (95% CI: £0.65, £0.76). The total cost was £13,792 in the “choice” arm and £10,690 in the “online first” arm. Hence, it cost an extra £47 to have one extra person to complete the questionnaire in the “choice” arm.

## 4 Discussion

### 4.1 Summary of main findings

In an RCT of two approaches to questionnaire completion in a population cohort study, we found that response rates were higher in the group offered a choice of online or paper questionnaire from the outset compared with those initially offered only online completion and that offering a choice of method cost an average of £0.71 more per participant than offering only online completion. The additional cost per completed questionnaire in the group offered a choice was £47.

### 4.2 What this adds to previous research

The effects of offering alternative methods of data collection have not been sufficiently evaluated in cohort studies [9]. There is a body of evidence about maximizing response rates to postal questionnaires in the survey literature. A Cochrane Review identified 110 methods of increasing response rates to postal questionnaires [10], many of which were found to improve response rates, including providing a second copy of the questionnaire at follow-up. A systematic review of cost-effectiveness of sample size maintenance programs in studies involving postal questionnaires revealed that insufficient economic information was reported to draw any general conclusions [26]. Edwards et al. [10] found that some of the methods found to increase response rates to postal questionnaires also applied to electronic questionnaires, and other methods were also found to be effective (including a statement that others had responded, lottery with immediate notification of results, and offer of survey results). For both paper and online questionnaires Edwards et al. [10] found substantial heterogeneity among trial results for half of the methods evaluated. This probably reflects the fact that “what works” is very context specific—depending on the type of the study, the country, the characteristics of the sample and how they were selected, and their expectations regarding study involvement.

The effectiveness of electronic questionnaires for data collection in cohort studies may differ from that in cross-sectional studies, and the use of online questionnaires as a primary data collection method in this setting has been relatively limited [12]. Nevertheless, they offer a potentially cost-effective way to collect data from participants and evidence from existing cohort studies in encouraging. The US Millenium Cohort Study of families associated with US Defense reported that, when given a choice, over 50% of participants chose to enroll online and that those who responded online provided more complete contact information [27]. An obvious drawback of electronic data collection is that not everyone has access to a computer, potentially introducing bias. In a study of Swedish women aged 30–49 years, Ekman et al. [28] assessed the feasibility of using online questionnaires in large population-based epidemiological studies. They concluded that the bias associated with using online questionnaires was not greater than that caused by paper questionnaires and that web-based questionnaires are a feasible tool for data collection in this setting. van Gelder et al. [29] summarize the advantages and disadvantages of online questionnaires for epidemiological studies and conclude that they could be considered an alternative or complementary mode of data collection, compared with the methods traditionally used by cohort studies, of paper questionnaires and face-to-face interviews.

It seems likely that a mixed-mode approach (i.e., using both paper questionnaires and online questionnaires) will be important in the gradual shift from paper to online data collection in cohort studies. Although there has been much debate in the survey literature on whether a concurrent or sequential mixed-mode approach is better [23], there is less evidence about this in the context of population cohort studies [19]. The YP in the ALSPAC study, born 1990–1991, are an ideal cohort on which to evaluate the effect of offering an online questionnaire first, compared with offering a choice of online or paper questionnaire completion. People of this age are highly mobile, and we anticipate that an online strategy will be key to maintaining their involvement in the future, but the implications for response rates of online data collection are not clear from the existing literature.

In this trial, we found that response rates were 10% lower among participants in the “online first” arm, compared with those offered a choice of online or paper completion.

### 4.3 Limitations

Due to attrition throughout the 21 years of the study, the cohort included in this study is not representative of the initial ALSPAC cohort or of the general population. The effects of attrition bias are documented elsewhere [14].

There were pragmatic differences between the two arms which reduced their comparability. For example, the questionnaire layout was not identical in the two methods (paper/online), and reminders and the compensatory gift voucher were not triggered in exactly the same way.

No complaints about the questionnaire or reminder process were received. However, a small number of participants (thirteen) reported having difficulties logging on to complete the questionnaire online. Considerable numbers contacted the study to report that they had lost their login details (256) or that never received them (115). Although login details were provided when such cases were reported, it is conceivable that these problems might have resulted in reducing the number of participants who chose to complete the questionnaire online, thereby possibly reducing the efficacy of the “online first” arm of the trial.

Another important limitation is that the invitation to complete the online questionnaire was by post rather than by email (which would make it easier to access). This was because, at the time of this questionnaire, email addresses were not routinely held for participants. As such contact details are updated, it will be possible to send out invitations by email, which may increase the number of people accessing the online questionnaire and will certainly reduce costs.

Finally, we note that this is a rapidly evolving field and that a limitation of any research in this field is the speed with which technology evolves. For example, the effectiveness of online data collection may be quite dependent on not only the proportion of the cohort who own a Smartphone but also on the functionality of online questionnaires when completed on Smartphones. It is a challenge for methodological research to keep up with new and emerging data collection tools [30].

### 4.4 Recommendations for further work

Future work should attempt to replicate research into optimal approaches to mixed-mode data collection published in the survey methodology literature in the context of cohort studies, where a more cautious approach to experimentation has been noted [19].

Further studies should assess the effectiveness of different types of reminders. In this study, the reminder method was dependent on the contact details available, but ideally, participants would be randomized to different reminder methods to assess their relative impact on response rates (see, e.g., [22]).

It is important to establish whether these results are generalizable to cohorts of other ages, such as the parents in the ALSPAC study. Future cohort studies could then tailor the contact method to different demographic groups.

## 5 Conclusions

Based on the findings of this RCT, embedded in a population cohort study, we conclude that there is some benefit in offering a choice of completion methods (concurrent mixed mode) compared with offering online-only first (sequential mixed mode) to maximize response rates to questionnaires. The results are likely to be generalizable to other cohorts of similar age and will help cohort studies weigh up the extra cost against anticipated improvements in response rates.

## Figures and Tables

**Table 1:** Reminder schedule for both arms of the trial

Time since initial letter	Reminder method
3 wks	Email/text/postcard reminder^a^
5 wks	Text/postcard reminder^b^ + Facebook reminder
8 wks	Letter with paper questionnaire
10 wks	Email/text/postcard reminder^a^ + Facebook reminder
12–19 wks	Phone call reminder

a Depending on contact details available.

b Depending on contact details available and not repeating the method used at 3 weeks.

**Table 2:** Numbers of questionnaires returned, by arm of trial

	Online first (*n* = 4,398)	Choice (*n* = 4,397)	Odds ratio (95% CI)	*P*-value	Adjusted odds ratio^a^ (95% CI)	*P*-value
Total number (%) of questionnaires returned	2,078 (47%)	2,144 (49%)	0.94 (0.87, 1.02)	0.16	0.90 (0.82, 0.99)	0.04

*Abbreviations:* CI, confidence interval; IMD, Index of Multiple Deprivation.

a Adjusted for gender, previous participation score (continuous), and IMD tertile.
