# Supplementary material for: Mode of delivery affected questionnaire response rates in a birth cohort study
Source: J Clin Epidemiol. 2017 Jan;81:64–71. doi: 10.1016/j.jclinepi.2016.09.004 (PMC5323059; doi:10.1016/j.jclinepi.2016.09.004)
Supplement: Supplementary Tables 1–4 [file mmc1.doc]

Supplementary Table 1. Numbers of each type of reminder in each arm of the trial
Online first (n=4398)	Choice (n=4397)

1st reminder - total
Email Text Postcard
2nd reminder - total
Text
Postcard
3378
771
539
2068
1214
585
629
3185
24
493
968
1114
498
616

3rd reminder (letter with paper questionnaire)	3242	2997

4th reminder - total
Email
Text
Postcard
2842
523
427
1892
2625
485
392
1748

5th reminder (phone call)	2003	1864


Supplementary Table 2. Completeness of questionnaires, by arm of trial

Online first (n=20371)
Choice
(n=21001)
Difference2
(95% CI)
p- value
Adjusted difference2,3 (95% CI)
p- value

Mean number of total questions completed4
Mean number of core questions completed4
190.4	193.6	3.20 (1.22,5.19)
100.8	101.3	0.47
(-0.33,1.27)
0.002	3.27 (1.28,5.25)
0.25	0.49
(-0.31,1.29)
0.001

0.23

1Number of YPs who completed at least one question, excluding those who used both methods of response
2Robust linear regression
3Ajusted for gender, previous participation (continuous) and IMD tertile
4Only questionnaires in which at least one question was completed are analysed


Supplementary Table 3. Mode of response, and requests for additional paper questionnaires, by arm of trial

Online first (n=4398)
Choice
(n=4398)
Difference1
(95% CI)
p- value
Adjusted difference1,2 (95% CI)
p- value

Responded by post	494	1106	0.38 (0.34,0.42)
Responded online	1601	1066	1.79 (1.63,1.96)
0.000	0.35 (0.31,0.39)
0.000	1.87 (1.70,2.06)
0.000

0.000

Requested additional paper copy
1Robust linear regression
121	80	1.53 (1.15,2.03)
0.004	1.53 (1.15,2.03)
0.004

2Ajusted for gender, previous participation (continuous) and IMD tertile


Supplementary Table 4. Costs for the 'choice' and 'online first' arms of the trial

Mean resource use	Mean cost (£)


Initial mailing:	Unit cost (£)	Choice
(n=4397)	Online first
(n=4398)	Choice
(n=4397)	Online first
(n=4398)	
admin time (per minute)	0.17	1.00	0.33	0.172	0.057	
red-bordered paper for letters (per sheet)	0.04	1.00	1.00	0.039	0.039	
labels for questionnaires (per label)
printing & stuffing questionnaire and
letters (per envelope)	0.008

0.43	1.00

1.00	0.00

0.00	0.008

0.430	0.000

0.000	
printing & stuffing letters (per envelope)	0.20	0.00	1.00	0.000	0.200	
posting (per envelope)	0.34	1.00	1.00	0.340	0.340	

1st reminder:						
Postcard						
printing (per card)	0.03	0.45	0.47	0.015	0.016	
admin time (per minute)	0.17	0.07	0.08	0.013	0.014	
postage (per card)	0.34	0.45	0.47	0.152	0.160	
Text						
sending text (per text)	0.04	0.11	0.12	0.004	0.004	

2nd reminder:						
Postcard						
printing (per card)	0.03	0.14	0.14	0.005	0.005	
admin time (per minute)	0.17	0.02	0.02	0.004	0.004	
postage (per card)	0.34	0.14	0.14	0.048	0.049	
Text						
sending text (per text)	0.04	0.11	0.13	0.004	0.005	

3rd reminder:						
Paper Questionnaire						
admin time (per minute)	0.17	0.68	0.74	0.117	0.127	
red-bordered paper for letters (per sheet)	0.04	0.68	0.74	0.027	0.029	
labels for questionnaires (per label)
printing & stuffing questionnaire and letters (per envelope)	0.008

0.43	0.68

0.68	0.74

0.74	0.005

0.293	0.006

0.317	
posting (per envelope)	0.34	0.68	0.74	0.232	0.251	

4th reminder:						
Text						
sending text (per text)	0.04	0.09	0.10	0.003	0.003	
Postcard						
printing (per card)	0.03	0.40	0.43	0.013	0.014	
admin time - casual staff (per min)	0.17	0.07	0.07	0.011	0.012	
postage (per card)	0.34	0.40	0.43	0.135	0.146	

5th reminder:						
Phonecall						
staff time - casual staff (per min)	0.17	1.27	1.37	0.219	0.235	

Reprinted questionnaire						
admin time (per min)	0.17	0.02	0.03	0.003	0.005	
red-bordered paper for letters (per sheet)	0.04	0.02	0.03	0.001	0.001	
labels for questionnaires (per label)
printing & stuffing questionnaire and letters (per envelope)	0.008

0.43	0.02

0.02	0.03

0.03	0.0001

0.008	0.002

0.012	
posting (per envelope)	0.34	0.02	0.03	0.006	0.009	


Return mail (per envelope)	0.34	0.25	0.11	0.086	0.038

Manual data entry of paper
questionnaires (per min)	0.17	4.31	1.93	0.743	0.332


Total mean cost (95% C.I's) per participant


Total cost(95% C.I's) per arm
3.14 (3.10-
3.18)

£13,791.61 (13619.51-
13963.72)
2.43(2.39-
2.47)

£10,690.11 (10,505.9-
10874.32)


Difference in adjusted total mean cost
(95% C.I's)	0.71(0.65-0.76)
